# Exercise Inhibits NLRP3 Inflammasome Activation in Obese Mice via the Anti-Inflammatory Effect of Meteorin-like

**DOI:** 10.3390/cells10123480

**Published:** 2021-12-09

**Authors:** Hafiz Muhammad Ahmad Javaid, Namood E Sahar, De-Li ZhuGe, Joo Young Huh

**Affiliations:** College of Pharmacy, Chonnam National University, Gwangju 61186, Korea; 186681@jnu.ac.kr (H.M.A.J.); sahar.namood@gmail.com (N.E.S.); zhugedeli@wmu.edu.cn (D.-L.Z.)

**Keywords:** exercise, myokines, adipose tissue, NLRP3 inflammasome, METRNL, type 2 diabetes

## Abstract

Obesity is associated with chronic low-grade inflammation. The benefits of exercise are partly attributed to its anti-inflammatory effect, but whether exercise can regulate NLRP3 inflammasome activation in obese adipose tissue remains unknown. Meteorin-like (METRNL), a recently discovered myokine, has been implicated in mediating the effect of exercise on metabolism. Herein, we examined the effect of exercise and METRNL on NLRP3 inflammasome activation. High-fat diet (HFD)-induced obese mice were subjected to treadmill exercise for 8 weeks. A subgroup of HFD mice was switched to normal chow with the exercise intervention. Exercise and diet attenuated weight gain, fat accumulation, and insulin resistance in obese mice. In addition, exercise downregulated gene and protein levels of inflammasome markers, including NLRP3 and caspase-1, in adipose tissue. In isolated bone marrow-derived macrophages, activation of NLRP3 inflammasome was suppressed in the exercise group, as confirmed by the downregulation of IL-1β and IL-18. Exercise significantly enhanced the expression of METRNL in various muscle depots, and further in vitro analysis revealed that recombinant METRNL treatment inhibited IL-1β secretion in macrophages. In conclusion, exercise exerts its anti-inflammatory action by suppressing adipose tissue NLRP3 inflammasome, and this is, in part, associated with METRNL induction in muscle and its anti-inflammatory effects in macrophages.

## 1. Introduction

Obesity has emerged as an epidemic worldwide, leading to an increase in metabolic disorders. Saturation of the expanding capacity of dysfunctional adipose tissue leads to lipid overflow and accumulation at various organs, leading to inflammation and insulin resistance [1]. In addition, inflammation in the adipose tissue itself, provoked by secretion of proinflammatory adipokines and recruitment of adipose tissue macrophages, has been linked to the development of obesity-induced metabolic dysregulations [2]. Specifically, activation of classical proinflammatory M1 macrophages and downregulation of anti-inflammatory M2 macrophages can exacerbate inflammation and cause insulin resistance in adipocytes [3]. Consequently, low-grade chronic inflammation in the adipose tissue can induce adipose tissue remodeling, leading to systemic effects associated with the increased risk of developing metabolic syndrome, type 2 diabetes, and cardiovascular diseases [4]. Therefore, it remains critical to clarify mechanisms involved in adipose tissue inflammation during obesity development to determine potential targets for the treatment of metabolic diseases.

The NLRP3 inflammasome is a key component of the innate immune system critical for host immune defense against bacterial, fungal, and viral infections [5,6,7]. However, recent studies have shown that the NLRP3 inflammasome is also involved in the molecular etiology of numerous chronic inflammatory diseases, including type 2 diabetes, non-alcoholic steatohepatitis, and heart failure [8,9]. In response to danger-associated molecular patterns, including saturated fatty acids, a sensor protein NLRP3 activates and recruits adaptor protein ASC, which binds with caspase-1. Caspase-1 mediates its inflammatory activity by cleavage and secretion of proinflammatory cytokines interleukin (IL)-1β and IL-18 [8,10]. Interestingly, IL-1β and IL-18 levels were found to be elevated in monocyte-derived macrophages isolated from patients with type 2 diabetes [11], and NLRP3, caspase-1, or IL-1β-deficient mice have reportedly exhibited improved insulin sensitivity [12], suggesting a major role of NLRP3 inflammasome in regulating glucose homeostasis [13].

Exercise is recognized as an effective non-pharmacological strategy that reduces the risk of metabolic and cardiovascular diseases [14,15]. In addition to benefits in terms of muscle strength and blood lipid levels, regular exercise can afford protective effects against inflammation. Reportedly, exercise can modulate the polarization of adipose tissue macrophages from M1 to M2 phenotype in high-fat diet (HFD)-induced obese mice [16,17]. Furthermore, exercise training has been shown to reduce the expression levels of toll-like receptors (TLRs) and proinflammatory cytokines in monocytes and adipose tissues, respectively [17,18]. Increased cytokine production and release from contracting skeletal muscles, termed as myokines, is regarded as one of the plausible underlying mechanisms for exercise-mediated anti-inflammatory effects [19,20]. It has been suggested that myokines can regulate the metabolic profile through crosstalk between muscle and adipose tissue by suppressing proinflammatory adipokine secretion from adipocytes during obesity [19,21]. However, whether exercise and exercise-induced myokines can regulate obesity-induced NLRP3 inflammasome activation in adipose tissue remains unclear.

Among various myokines, we focused on the role of Meteorin-like (METRNL). METRNL is produced upon stimulation by exercise and promotes adipose tissue browning via alternative macrophage induction at adipose depots [22]. Further research revealed that METRNL plays a protective role in cardiac dysfunction and lipid-induced muscle insulin resistance by ameliorating inflammation [23,24]. In the present study, we used HFD-induced obese mice and isolated bone marrow-derived macrophages (BMDMs) to examine how exercise and METRNL regulate the activation of NLRP3 inflammasome. Our results revealed that exercise inhibits NLRP3 inflammasome activation in subcutaneous and visceral adipose tissue, as well as in BMDMs. Moreover, we observed that METRNL stimulation suppressed NLRP3 inflammasome activation and subsequent IL-1β secretion in part by stimulating the extracellular signal-regulated kinase (ERK) and p38 mitogen-activated protein kinase (MAPK) pathway. Collectively, these findings suggest a novel role of exercise and METRNL in the regulation of inflammation during obesity.

## 2. Materials and Methods

### 2.1. Animal Studies

C57BL/6 male mice (Orient Bio Inc.; Seongnam, South Korea) were housed at 22 ± 2 °C, 50–60% humidity, and under a 12 h light/12 h dark cycle in a pathogen-free room. Water and food were provided *ad libitum* throughout the study period. Six-week-old mice were randomly divided by weight into two groups, fed either a normal chow diet (NCD) or an HFD. The NCD mice were fed a standard diet composed of 4.5% fat (3.41 kcal/g); HFD mice were fed a diet containing 60% fat, 21.3% carbohydrate, and 18.4% protein (5.24 kcal/g, Research Diets, Inc.; New Brunswick, NJ, USA). After six weeks, mice fed the HFD were randomized by weight to one of the following three groups: (1) HFD, an obese control group that maintained HFD; (2) HFD+E, continuation of HFD with exercise; (3) HFNCD+E, mice switched to NCD with exercise (*n* = 8 per group). The exercise program consisted of 8 weeks of treadmill running during their active cycle, 5 days/week starting at 12 m/min for 20 min/day in the first week; this was followed by up to 20 m/min for 50 min/day in the last week with no electric shock and 0° slope. The exercise training included a 5 min warm-up/cool-down period. For adapting mice to the exercise protocol, a change in diet in the HFNCD+E group was performed 2 weeks after starting the exercise regimen. Food intake was determined by measuring the difference between the pre-weighed chow provided and the remaining food at the end of each week for a period of three weeks before sacrifice. Oral glucose tolerance test (OGTT) and intraperitoneal insulin tolerance test (IPITT) were performed at week 8 of the exercise training program. For OGTT, mice were fasted for 12 h during their active cycle and administered an oral dose of 2 g glucose/kg body weight. IPITT was performed after 6 h of fasting during the active cycle, followed by an intraperitoneal injection of 1 U/kg body weight insulin (I0516, Sigma; St. Louis, MO, USA). For both OGTT and IPITT, blood glucose was measured from the tail vein using a glucometer (Accu-Check Performa, Roche; Basel, Switzerland) at respective time points. For IPITT results, inverse AUC was calculated to correct for the difference in baseline glucose levels. Briefly, basal glucose levels (time point 0) were subtracted from all later obtained blood glucose levels for each mouse individually. The values were inverted (multiplication with −1), followed by the calculation of the individual AUCs. Mice were sacrificed 24 h after the end of the exercise program at 20 weeks of age. Animals at a fed state were anesthetized with isoflurane and perfused with phosphate-buffered saline (PBS). Then, epididymal fat, subcutaneous fat, brown fat, gastrocnemius muscle, quadriceps femoris muscle, soleus muscle were excised, weighed, and immediately stored at −80 °C. Animal experiments were approved by the Institutional Animal Care and Use Committee (IACUC) of Chonnam National University.

### 2.2. Enzyme-Linked Immunosorbent Assays

Immediately after sacrifice, blood was collected via intracardiac puncture for plasma analysis, centrifuged for 15 min at 3000 rpm (842 rcf) at 4 °C, and stored at −80 °C until further use. Plasma levels of IL-1β (MLB00C, R&D system), insulin (80-INSMS-E01, ALPCO; Salem, NH, USA), and adiponectin (MRP300, R&D system; Minneapolis, MN, USA) were measured using ELISA kits, according to the manufacturer’s instructions. In addition, IL-1β (88-7013-88, Invitrogen; Carlsbad, CA, USA) and IL-18 (7625, MBL Life Science; Nagoya, Japan) levels were measured in BMDM supernatants. Tissue lysates from different muscle depots were used to measure METRNL (DY6679, R&D system) levels in accordance with the manufacturer’s instructions.

### 2.3. Free Fatty Acids and Triglycerides

Plasma-free fatty acids (FFA) and triglycerides were measured using FFA (ab65341, Abcam; Cambridge, UK) and triglyceride (ab65336, Abcam) assay kits. The optical density was measured at 570 nm, and data analysis was performed according to the manufacturer’s instructions.

### 2.4. Macrophage Count and Immunohistochemistry

After harvesting, adipose tissues were fixed in 10% neutral buffered formalin, dehydrated, cleared, and paraffin-embedded. Then, paraffin blocks were cut to 4 μm thick sections. Hematoxylin-eosin (H&E) staining was performed using Dako CoverStainer (Agilent, Santa Clara, CA, USA). For examining macrophage infiltration, sections were stained with F4/80 antibodies (1:200; Abcam, Cambridge, UK) and then labeled using polymer Dako EnVision+ System-HRP (Agilent; Santa Clara, CA, USA), in accordance with the manufacturer’s instructions. After staining, sections were scanned with a Pannoramic SCAN II scanner (3DHISTECH Kft., Budapest, Hungary). Adipocyte size and the number of macrophages in the adipose tissue were quantified using ImageJ (NIH, Bethesda, MD, USA).

### 2.5. Isolation and Culture of Bone Marrow-Derived Macrophages

Briefly, mice were sacrificed via cervical dislocation. Then, the tibia and fibula were harvested, and bone marrow cells were collected. After centrifugation for 1 min at 10,000 rpm (9358 rcf), cells were seeded in plates and incubated for 7 days using Iscove’s Modified Dulbecco’s Medium (Gibco, Carlsbad, CA, USA), supplemented with 10% fetal bovine serum (FBS; HyClone; Logan, UT, USA), 30% L929-conditioned media, 1% penicillin/streptomycin (Gibco; Carlsbad, CA, USA), 1% sodium pyruvate (Gibco), and 1% MEM non-essential amino acids (Gibco). BMDMs were primed for 6 h with 100 ng/mL lipopolysaccharide (LPS; L2630, Sigma), followed by 2 mM ATP (A6419, Sigma) treatment for the final 30 min before harvesting.

For the conditioned media experiment, BMDMs at day 7 were washed with PBS, and fresh media was added and then collected after 24 h. The collected media was centrifuged at 13,000× *g* rpm (15,814 rcf) for 10 min to remove debris and stored at −80 °C until the treatment of 3T3-L1 mature adipocytes using this conditioned media for 24 h.

### 2.6. 3T3-L1 Cell Culture and Treatment

3T3-L1 mouse pre-adipocytes were purchased from ATCC (Manassas, VA, USA). The cells were maintained in Dulbecco modified Eagle medium (DMEM) with 10% FBS and 1% penicillin/streptomycin and incubated at 37 °C in 5% CO_2_. Pre-adipocytes were differentiated as previously described [25]. Briefly, two days after 3T3-L1 pre-adipocytes reached 100% confluency, cells were treated with differentiation media containing insulin, isobutylxanthine, dexamethasone, and rosiglitazone. To examine the effects of METRNL, mature 3T3-L1 adipocytes were treated with 100 ng/mL recombinant METRNL (AG-40B-0149) on day 8 for 1 h, followed by 10 ng/mL tumor necrosis factor (TNF)-α (T7539, Sigma) for 24 h.

### 2.7. Gene Expression Analysis

Total RNA was extracted from cells and animal tissues using TRI Reagent (MRC, Cincinnati, OH, USA). cDNA was synthesized using TOPscript^TM^ RT DryMIX (Enzynomics; Daejeon, South Korea). mRNA levels were measured by real-time PCR using Rotor-Gene Q (QIAGEN; Hilden, Germany) with 20 μL reaction volume consisting of cDNA transcripts, primer pairs, and TOPreal SYBR Green PCR Kit (Enzynomics). The gene expression levels were normalized to 18S rRNA levels.

### 2.8. Western Blot Analysis

Briefly, tissue samples and cells were lysed with RIPA buffer (Thermo Scientific, Rockford, IL, USA). After quantification using the BCA kit (Thermo Scientific; Rockford, IL, USA), 50 μg of protein sample was separated by SDS-PAGE and transferred to PVDF membranes. After blocking for 1 h with 5% skim milk, membranes were incubated with primary antibodies at 4 °C overnight. After overnight incubation, membranes were incubated with the secondary antibodies at room temperature for 2 h, and proteins on the blots were detected with LAS-4000 (Fuji Photo Film, Tokyo, Japan). NLRP3 (#1510, 1:2000), p62 (#5114, 1:2000), adiponectin (#2789, 1:2000), β-tubulin (#2146, 1:2000), phospho-ERK (#9101, 1:2000), total-ERK (#9102,1:2000), phospho-p38 (#4631,1:2000), and total-p38 MAPK (#8690,1:2000) antibodies were purchased from Cell Signaling Technology (Danvers, MA, USA). Caspase-1 (#sc-56036, 1:500), β-actin (#sc-47778, 1:2000), mouse anti-rabbit IgG-HRP (#sc-2357, 1:2000), and m-IgGƙ BP-HRP (#sc-516102, 1:2000) were procured from Santa Cruz Biotechnology Inc (Dallas, TX, USA).

### 2.9. Statistical Analysis

Data analyses were performed using Statview v5.0 software (SAS Institute Inc., Cary, NC, USA). Data are expressed as the mean ± standard error of the mean (SEM). Mean values obtained from each group were compared using one-way ANOVA, followed by Fisher’s Protected Least Significant Difference (PLSD) *post-hoc* test. A *p*-value < 0.05 was used to establish statistical significance.

## 3. Results

### 3.1. Exercise and Diet Attenuate Weight Gain and Fat Accumulation in HFD-Induced Obese Mice

**Figure 1:**
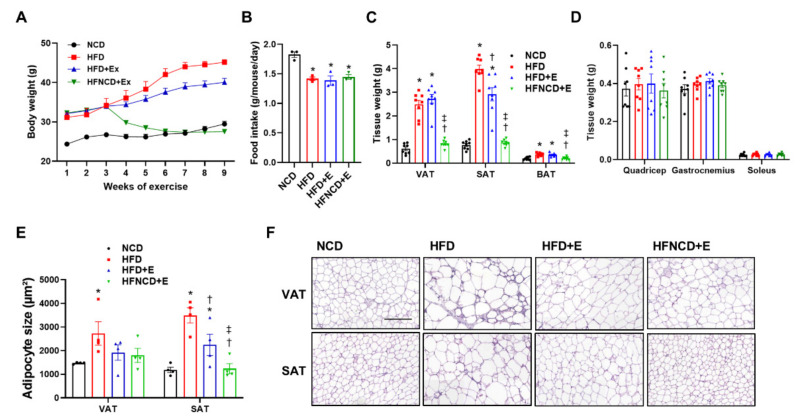
Exercise and diet attenuate weight gain and fat accumulation in HFD-induced obese mice. (**A**) Body weight change throughout the exercise intervention was measured. (**B**) Daily food intake was calculated per mouse on average for three weeks before sacrifice. (**C**,**D**) Tissues isolated after sacrifice were weighed. (**E**,**F**) H&E staining was performed for adipocyte morphology, and average adipocyte size was measured (scale bar indicates 200 µm). Values are presented as mean ± SEM. * *p* < 0.05 compared with NCD, † *p* < 0.05 compared with HFD, ‡ *p* < 0.05 compared with HFD+E.

To observe the effect of exercise on body composition and metabolism, HFD-induced obese mice were subjected to an eight-week treadmill exercise regimen. In a subgroup of mice, HFD was switched to NCD to determine the combined effect of exercise and diet. Prior to initiating the exercise regimen, the body weight was significantly increased in HFD mice when compared with NCD mice (Figure 1A). In HFD+E mice, body weight was significantly decreased five weeks after initiating the exercise intervention. In contrast, body weight was immediately reduced in the HFNCD+E group within one week of switching the diet when compared with both HFD and HFD+E groups. The change in body weight among groups was independent of food consumption (Figure 1B). At the end of the study period, the HFD group showed a significant increase in visceral adipose tissue (VAT) and subcutaneous adipose tissue (SAT) when compared with the NCD group (Figure 1C). Interestingly, VAT was decreased in the HFNCD+E mice but not in HFD+E mice, whereas SAT was significantly decreased in both HFD+E and HFNCD+E mice when compared with HFD controls. In both WAT depots, a combination of exercise and diet was more effective than exercise alone, which led to a considerable reduction in tissue weight similar to that observed in the NCD group. Brown adipose tissue (BAT) was also significantly increased in HFD and HFD+E groups and decreased in HFNCD+E mice. There was no evident change in weight for different muscle types harvested (Figure 1D). On assessing adipose tissue morphology, we observed that adipocyte size was reduced in the HFD+E group in both VAT and SAT, although only statistically significant in SAT (Figure 1E,F). Similar to tissue weight, adipocytes were smaller in the SAT of the HFNCD+E group when compared with that of the HFD+E group. Overall, these results indicate that exercise reverses the HFD-induced increase in body weight, adipose tissue mass, and adipocyte hypertrophy, demonstrating a more pronounced effect in combination with diet.

### 3.2. Exercise and Diet Ameliorates Metabolic Dysregulation in HFD-Induced Obese Mice

**Figure 2:**
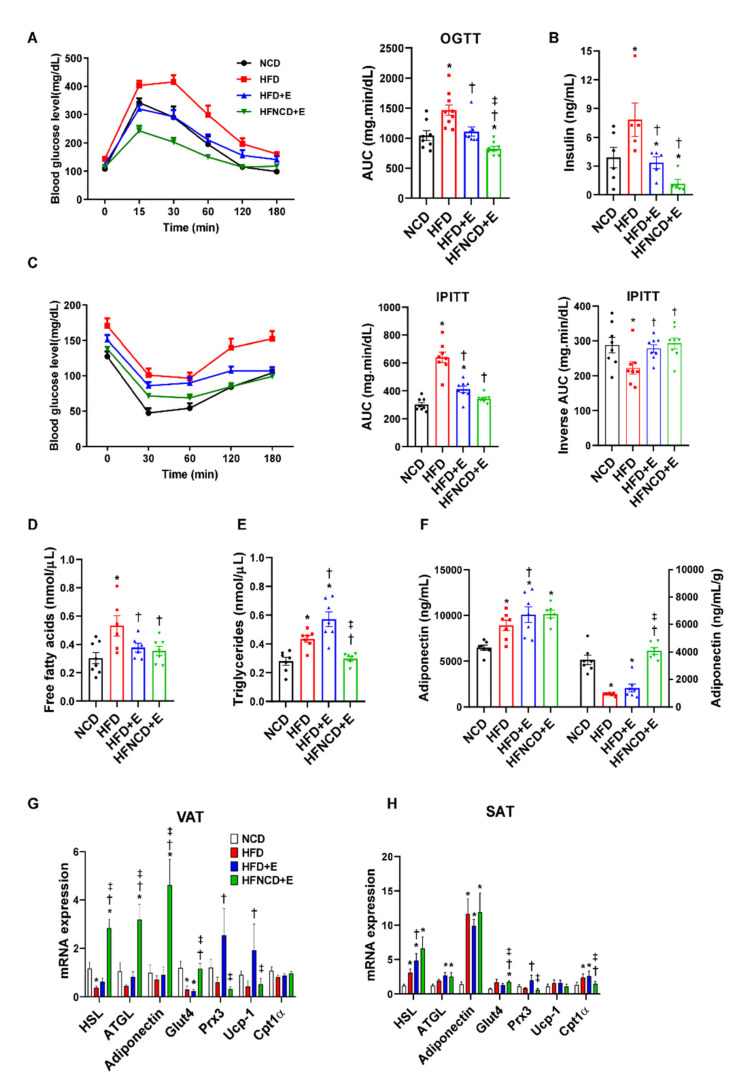
Exercise and diet ameliorate metabolic dysregulation in HFD-induced obese mice. (**A**) Oral glucose tolerance test (OGTT) and (**C**) intraperitoneal insulin tolerance test (IPITT) were performed at week 8 of the exercise training program. Blood glucose levels were measured at 0, 30, 60, 120, and 180 min, and the AUC was determined. For IPITT, inverse AUC was also calculated, correcting for baseline glucose levels. (**B**) Plasma insulin, (**D**) free fatty acid, (**E**) triglyceride, and (**F**) adiponectin levels were measured by ELISA in 20-week-old mice sacrificed in fed state, 24 h after the end of the exercise program. Plasma adiponectin levels (left panel in F) were adjusted by adipose tissue weight (right panel). (**G**,**H**) Metabolic gene expression was measured in visceral and subcutaneous adipose tissues via real-time PCR. The quantifications were normalized to the 18S rRNA level for each target. Values are presented as mean ± SEM. * *p* < 0.05 compared with NCD, † *p* < 0.05 compared with HFD, ‡ *p* < 0.05 compared with HFD+E.

To assess the effect of exercise and diet on glucose homeostasis, we conducted an oral glucose tolerance test. As a result, HFD mice developed impaired glucose tolerance when compared with NCD mice, which was significantly reversed by exercise intervention (Figure 2A). Exercise in combination with diet afforded an additive effect and elicited improved glucose levels when compared with both HFD and HFD+E groups. Similarly, IPITT results revealed that insulin resistance was significantly ameliorated following exercise in HFD mice and further improved when combined with diet (Figure 2C). Since baseline glucose levels were different among groups, IPITT results were further calculated for inverse AUC values. The baseline-corrected inverse AUC was lower in the HFD-fed mice compared to control mice, suggesting decreased insulin sensitivity, which was reversed in both exercise groups. Furthermore, plasma insulin levels showed a similar trend (Figure 2B). The plasma lipid profile revealed that HFD upregulated both FFA and triglyceride levels (Figure 2D,E). In contrast, FFA levels were downregulated in both HFD+E and HFNCD+E groups, whereas triglyceride levels were significantly downregulated in the HFNCD+E group. Adiponectin is an anti-inflammatory adipokine, and its circulating levels reflect whole-body insulin sensitivity [26,27]. In the present study, plasma adiponectin levels were significantly elevated in all HFD-fed groups when compared with the NCD group (Figure 2F). However, when adjusted by fat mass, the levels were reduced in HFD compared to NCD mice, implying a relative deficiency in adiponectin secretion per gram of adipose tissue [28]. The decreased levels by HFD were only restored in the HFNCD+E group but not in the HFD+E group. These results indicate that exercise alone could effectively ameliorate HFD-induced impairment at the systemic level, while diet combined with exercise elicits an additive effect.

Next, mRNA expression levels of metabolic markers were measured to further evaluate the effect of exercise and diet on adipocyte metabolism. In VAT of HFD mice, hormone-sensitive lipase (HSL) and glucose transporter type 4 (Glut4) mRNA levels were significantly decreased, whereas mRNA expression levels of adipose triglyceride lipase (ATGL), adiponectin, peroxiredoxin 3 (Prx3), and uncoupling protein 1 (UCP1) showed a decreasing trend when compared with NCD mice (Figure 2G). Among downregulated markers, HSL, ATGL, adiponectin, and Glut4 levels were significantly elevated in the HFNCD+E group, whereas Prx3 and UCP1 levels were increased in the HFD+E group. Gene expression in SAT demonstrated a different trend, where mRNA expression levels of HSL, ATGL, and adiponectin were elevated in all HFD-fed groups (Figure 2H). Similar to VAT, Glut4 levels were increased in the HFNCD+E group, and Prx3 was elevated in the HFD+E group. These findings partly explain the differences in weight changes in VAT and SAT observed in HFD mice in response to exercise and diet.

### 3.3. Exercise and Diet Ameliorate HFD-Induced Inflammation and Fibrosis in Adipose Tissues

**Figure 3:**
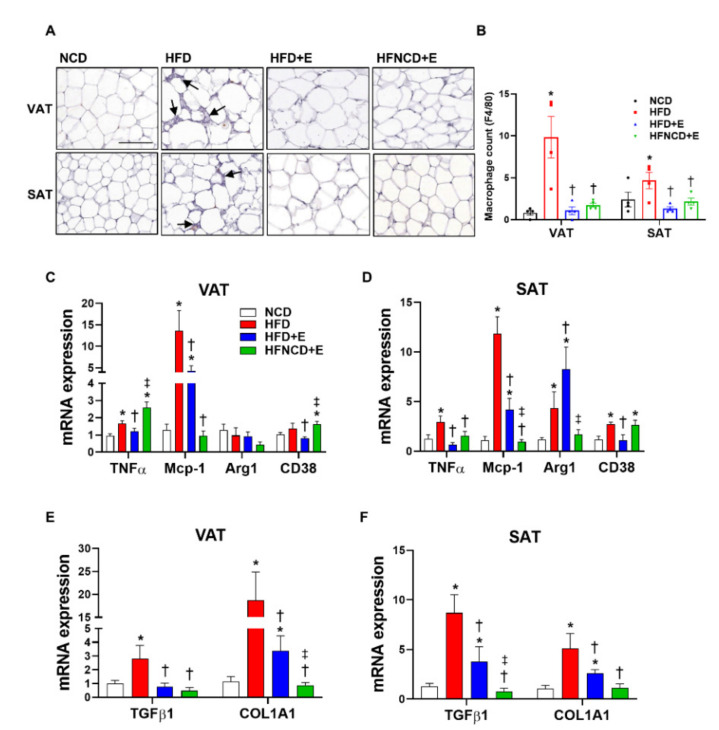
Exercise and diet ameliorate HFD-induced inflammation and fibrosis in adipose tissue. (**A**,**B**) Adipose tissues were stained with F4/80 and positive cells were counted as infiltrated macrophages (arrows indicate macrophages, scale bar indicates 100 µm). Inflammatory gene expression (**C**,**D**) and fibrosis markers (**E**,**F**) were measured in visceral and subcutaneous adipose tissues by real-time PCR. The quantifications were normalized to the 18S rRNA level for each target. Values are presented as mean ± SEM. * *p* < 0.05 compared with NCD, † *p* < 0.05 compared with HFD, ‡ *p* < 0.05 compared with HFD+E.

Inflammation in adipose tissue is considered a major factor that mediates the pathophysiology of obesity-induced metabolic dysregulation. Assessment of macrophage infiltration by F4/80 staining showed significant upregulation in both VAT and SAT in HFD-fed mice (Figure 3A). Interestingly, in both adipose depots, exercise alone sufficiently suppressed macrophage infiltration. Exercise downregulated the number of infiltrated macrophages similar to those in NCD mice, and no further reduction was observed in mice following the dietary intervention (Figure 3B). To further evaluate the inflammatory status in adipose tissues, mRNA levels of proinflammatory markers were measured. In both adipose depots, HFD-induced TNF-α and monocyte chemoattractant protein-1 (MCP-1) levels were significantly downregulated by exercise (Figure 3C,D). Whereas MCP-1 levels were further downregulated in combination with diet, HFD-induced TNF-α levels in VAT were not suppressed in the HFNCD+E group when compared with HFD controls. Similarly, mRNA expression of CD38, a marker of M1 macrophage polarization, was suppressed in the HFD+E group when compared with the HFD group, but this effect was not observed in the HFNCD+E group in both depots. Expression of arginase 1 (Arg1), a marker of M2 macrophage polarization, was induced by HFD in SAT and was further increased in the HFD+E group when compared with the NCD group. This increase was not observed in the HFNCD+E group. These results suggest that exercise alone could ameliorate HFD-induced adipose tissue inflammation in both VAT and SAT through distinct patterns in adipose depots.

Recent reports have emphasized the role of adipose tissue fibrosis in exacerbating adipose tissue inflammation [29]. However, the role of exercise in adipose tissue fibrosis remains to be further elucidated. Figure 3E,F show that upregulated mRNA expression levels of transforming growth factor (TGF)-β1 and collagen type 1, alpha 1 (COL1A1) in HFD mice were significantly downregulated by exercise alone and were further suppressed on combining exercise and diet.

### 3.4. Exercise and Diet Attenuate HFD-Induced NLRP3 Inflammasome Expression in Adipose Tissues

**Figure 4:**
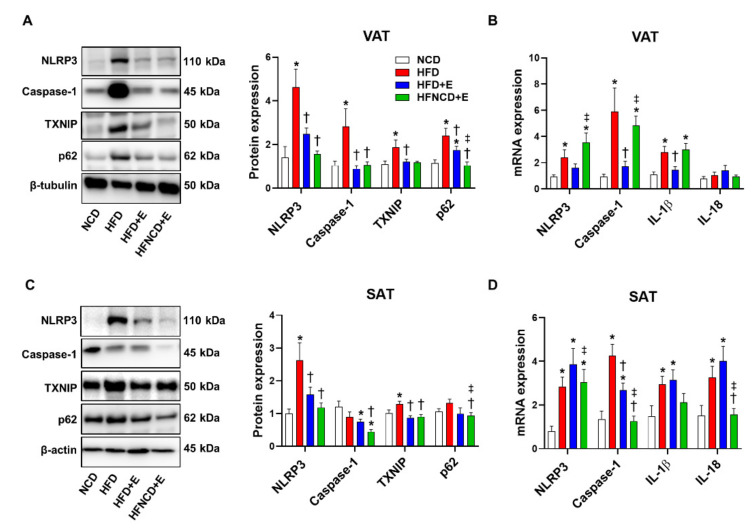
Exercise and diet attenuate HFD-induced NLRP3 inflammasome expression in adipose tissues. Representative Western blots and quantifications in VAT (**A**) and SAT (**C**) lysates, respectively. The quantifications were normalized to either β-tubulin (VAT) or β-actin (SAT) levels. Gene expressions were measured using real-time PCR in VAT (**B**) and SAT (**D**). The quantifications were normalized to the 18S rRNA level for each target. Values are presented as mean ± SEM (*n* = 8). * *p* < 0.05 compared with NCD, † *p* < 0.05 compared with HFD, ‡ *p* < 0.05 compared with HFD+E.

Next, we examined the effect of exercise and diet on HFD-induced NLRP3 inflammasome activation in adipose tissues. Accordingly, NLRP3 inflammasome components were measured in VAT and SAT. In HFD controls, NLRP3 inflammasome activation was detectable when compared with the NCD group, as indicated by increased protein and mRNA levels of NLRP3 and caspase-1 in both VAT and SAT (Figure 4).

In VAT, exercise decreased protein levels of NLRP3 and caspase-1 to a similar extent with or without diet (Figure 4A). In addition, exercise significantly downregulated upstream inducers of the NLRP3 inflammasome, i.e., TXNIP and p62. In addition, mRNA expression levels of NLRP3, caspase-1, and IL-1β were downregulated in the HFD+E group when compared with HFD controls; however, exercise combined with dietary intervention reversed the effect of exercise and aggravated the inflammasome marker expression (Figure 4B).

In SAT, exercise alone or combined with diet intervention decreased protein levels of NLRP3 and TXNIP, similar to the observations in VAT (Figure 4C). Caspase-1 and p62 protein expression were not significantly upregulated by HFD, but their levels were significantly lower in the HFNCD+E group. The cleaved form of caspase-1 was not detected in either VAT or SAT. Gene expression levels of NLRP3, caspase-1, IL-1β, and IL-18 were upregulated in the HFD group when compared with the NCD group. Among them, mRNA expression levels of only caspase-1 were reduced in the HFD+E group when compared with HFD mice and decreased further in the HFNCD+E group (Figure 4D). HFD-induced NLRP3 and IL-18 mRNA expression was significantly decreased in the HFNCD+E group. Despite significant downregulation in mRNA and protein expression of NLRP3 inflammasome in response to exercise and calorie restriction, plasma IL-1β levels did not differ among groups (Figure S1). In summary, exercise alone or combined with diet attenuated the HFD-induced NLRP3 inflammasome expression in adipose tissues.

### 3.5. Exercise and Diet Attenuate HFD-Induced NLRP3 Inflammasome Activation in BMDMs

**Figure 5:**
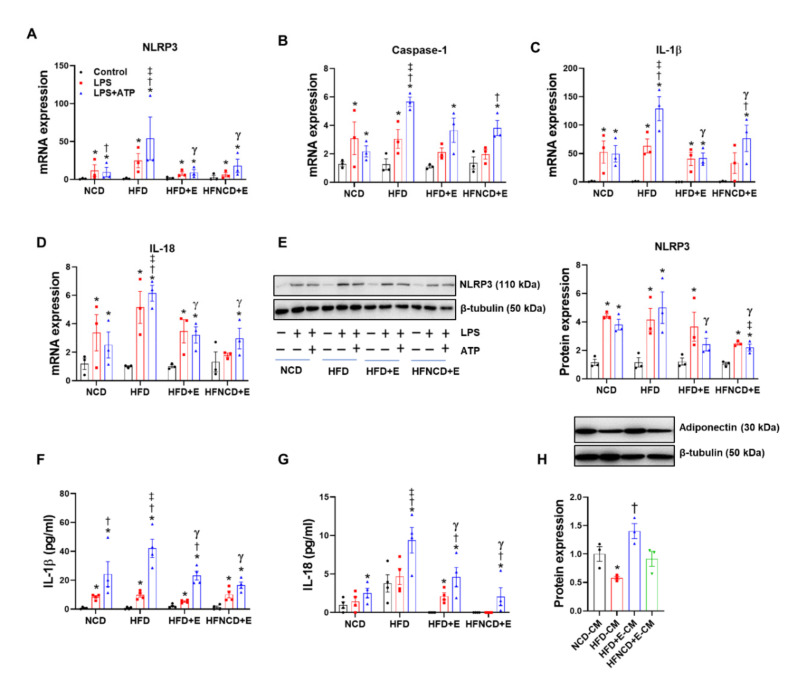
Exercise and diet attenuate HFD-induced NLRP3 inflammasome activation in BMDMs. (**A**–**D**) BMDMs isolated from experimental mice were treated with LPS (100 ng/mL) for 6 h and ATP (2 mM) in the last 30 min. Gene expression levels were measured using real-time PCR, and the quantifications were normalized to the 18S rRNA level for each target. (**E**) Representative Western blots and quantification of NLRP3 in BMDMs isolated from experimental mice. (**F**,**G**) IL-1β and IL-18 were measured in the BMDM supernatants using ELISA. (**H**) Representative Western blots and quantification of adiponectin in 3T3-L1 adipocytes treated for 24 h with conditioned media obtained from BMDMs isolated from each group. The protein levels were normalized to β-tubulin. Values are presented as mean ± SEM. * *p* < 0.05 compared with control, † *p* < 0.05 compared with LPS, ‡ *p* < 0.05 compared with NCD LPS+ATP, γ *p* < 0.05 compared with HFD LPS+ATP.

Although we observed that activation of NLRP3 occurs in adipose tissues of obese mice, the precise cell type mediating this response remains unclear, as adipose tissue is comprised of various types of cells [30]. It can be postulated that this inflammatory signal primarily involves immune cells. As isolating infiltrated macrophages from adipose tissue can be challenging, as an alternative, we isolated BMDMs from each mouse group and prepared ex vivo cultures to observe whether the effect of HFD and exercise on NLRP3 inflammasome in adipose tissue can be replicated. In BMDMs isolated from HFD-fed control mice, LPS and ATP stimulation amplified the NLRP3 inflammasome expression, which is the priming step (signal 1), when compared with NCD mice, as indicated by increased mRNA levels of NLRP3, caspase-1, IL-1β, and IL-18 (Figure 5A–D). The protein level of NLRP3 showed a trend toward upregulation in response to LPS and ATP in the HFD group when compared with the NCD group; however, this effect was not significant (Figure 5E). Interestingly, the effect due to exercise alone was sufficient to significantly downregulate mRNA and protein expression levels of NLRP3 inflammasome markers induced by LPS and ATP. Moreover, the reduced response was comparable in BMDMs isolated from HFD+E and HFNCD+E groups. To confirm the effect on the activation step (signal 2), secretion of IL-1β and IL-18 in BMDM culture media was measured via ELISA. LPS and ATP treatment significantly upregulated both IL-1β and IL-18 levels in BMDMs from HFD controls when compared with those from the NCD group; both levels were successfully downregulated by exercise, with or without dietary intervention (Figure 5F,G). These results indicate that exercise could regulate macrophage function by suppressing NLRP3 inflammasome activation.

Crosstalk between adipocytes and infiltrated macrophage in adipose tissue plays an important role in the development of obesity [31]. Accordingly, 3T3-L1 mature adipocytes were treated with conditioned media from cultured BMDMs isolated from each mouse group. The conditioned media from HFD control BMDMs significantly downregulated adiponectin protein expression in adipocytes. Moreover, downregulated adiponectin levels were recovered following treatment with conditioned media from the HFD+E group BMDMs, whereas the conditioned media from the HFNCD+E group BMDMs had no effect (Figure 5H), confirming that exercise-mediated regulation of macrophage NLRP3 inflammasome activation could be involved in normalizing energy homeostasis in adipocytes, partly by restoring adiponectin expression.

### 3.6. Exercise and Diet Induce METRNL in Different Muscle Depots of HFD-Induced Obese Mice

**Figure 6:**
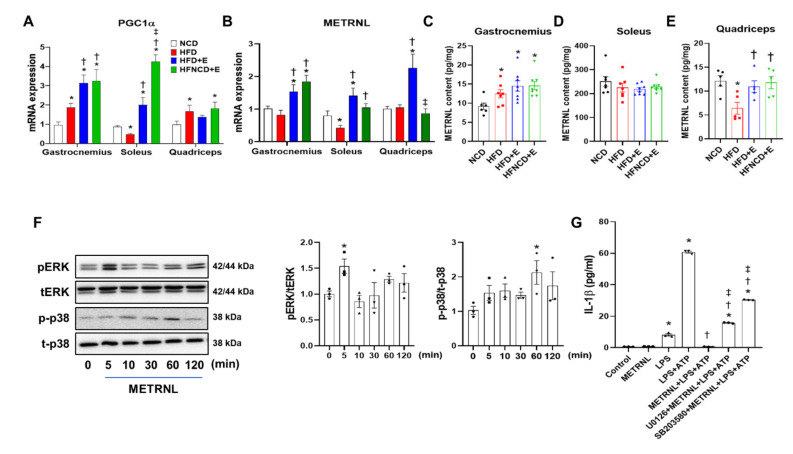
METRNL is induced by exercise in muscle and inhibits NLRP3 inflammasome activation in BMDMs. Gene expression levels of PGC1α (**A**) and METRNL (**B**) in the gastrocnemius, soleus, and quadriceps muscles were measured in mice using real-time PCR and normalized to 18S rRNA levels. (**C**–**E**) METRNL protein content in soleus, gastrocnemius, and quadriceps was measured using ELISA. (**F**) Representative Western blots and quantification showing time-dependent activation of ERK and p38 MAPK signaling in METRNL-treated (100 ng/mL) BMDMs. BMDMs were isolated from 5-week-old male C57BL/6 mice. The phosphorylated protein levels were normalized to total protein levels (p = phosphorylated, t = total) (**G**) BMDMs were treated with U0126 (30 μM) and SB203580 (30 μM) for 1 h, followed by METRNL (100 ng/mL) for 1 h and LPS+ATP treatment for 6 h. The supernatant was collected, and IL-1β was measured using ELISA. Values are presented as mean ± SEM. * *p* < 0.05 compared with NCD, † *p* < 0.05 compared with HFD, ‡ *p* < 0.05 compared with HFD+E (**A**–**E**). Values are presented as mean ± SEM of triplicate. * *p* < 0.05 compared with control, † *p* < 0.05 compared with LPS+ATP, ‡ *p* < 0.05 compared with METRNL+LPS+ATP (**F**–**G**).

Reportedly, exercise-induced myokines exert their anti-inflammatory effect via muscle-adipose tissue crosstalk [19]. Among various myokines discovered to date, we examined the expression of METRNL in the gastrocnemius, soleus, and quadriceps femoris muscles. In HFD-induced obese mice, the mRNA level of METRNL was downregulated in the soleus muscle, whereas it remained stable in all other muscle types. In contrast, METRNL mRNA levels were significantly upregulated in the HFD+E group when compared with the NCD or HFD groups in all three muscle types (Figure 6B). In the gastrocnemius and soleus muscles, increased METRNL mRNA levels were comparable between exercise groups, with or without dietary intervention. However, this increase was not detected in the quadriceps muscles of HFNCD+E mice. The mRNA expression of PGC1α, which is a transcriptional coactivator that mediates the benefits of exercise [32], showed a similar trend with METRNL mRNA in gastrocnemius and soleus but not quadriceps muscle (Figure 6A). Next, we examined the protein levels of METRNL and observed that the amount of METRNL per milligram tissue was highest in the soleus muscle (Figure 6C–E). The gastrocnemius muscle of all HFD-fed groups exhibited significantly higher levels of METRNL than that of NCD mice (Figure 6C). Despite higher basal expression levels, no changes were observed in the soleus muscle among groups (Figure 6D). In the quadriceps muscle, METRNL levels were significantly downregulated in the HFD group, which were successfully recovered by exercise with or without dietary intervention (Figure 6E). Although variation among different muscle depots exists, these results indicate that exercise alone or in combination with diet could upregulate the METRNL levels at both mRNA and protein levels.

### 3.7. METRNL Inhibits NLRP3 Inflammasome Activation in BMDMs through ERK and P38 MAPK Signaling

Next, we evaluated whether exogenous METRNL treatment could suppress NLRP3 inflammasome activation in BMDMs. Accordingly, pretreatment with recombinant METRNL completely abolished IL-1β secretion induced by LPS and ATP in BMDMs (Figure S2). In addition, to determine signaling pathways through which METRNL exerts its anti-inflammatory effects, we performed METRNL treatment at different time points and examined the phosphorylation of ERK and p38 MAPK. Based on our findings, METRNL significantly upregulated the expression of phosphorylated ERK and p38 MAPK at 5 min and 1 h, respectively (Figure 6F). ERK inhibitor (U0126) and p38 MAPK inhibitor (SB203580) were employed to further confirm whether these pathways participated in ameliorating inflammation (Figure S3). Of note, both ERK and p38 MAPK inhibitors were effective in reducing IL-1β secretion induced by LPS and ATP (Figure S4), implying the anti-inflammatory action of these inhibitors. In contrast, the inhibitory effect of METRNL on IL-1β secretion was significantly reversed by both inhibitors, and IL-1β levels were restored to some extent (Figure 6G). These results indicate that both ERK and p38 MAPK pathways are activated by METRNL and in part mediate its anti-inflammatory effect in BMDMs.

### 3.8. METRNL Ameliorates Gene Expression but Not Protein Expression of TNF-α-Induced NLRP3 Inflammasome in Cultured Adipocytes

**Figure 7:**
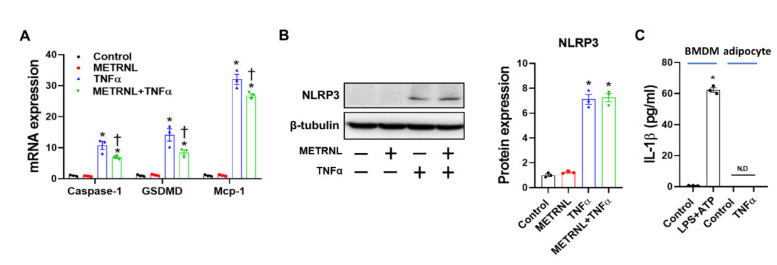
METRNL inhibits gene expression but not protein expression of NLRP3 inflammasome in 3T3-L1 adipocytes. (**A**) Mature 3T3-L1 adipocytes were pretreated with METRNL (100 ng/mL) for 1 h, followed by treatment with TNF-α (10 ng/mL) for 24 h. Gene expression levels were measured via real-time PCR. The quantifications were normalized to the 18S rRNA level for each target. (**B**) Representative Western blots and quantification of NLRP3 in mature adipocytes. (**C**) Mature adipocytes were treated with TNF-α (10 ng/mL) for 24 h, and IL-1β levels in supernatant were measured using ELISA. BMDMs treated with LPS+ATP for 6 h are shown as a positive control. Values are presented as mean ± SEM. * *p* < 0.05 compared with control, † *p* < 0.05 compared with TNF-α.

Next, we assessed whether the effect of METRNL on inflammasome involves not only macrophages but also adipocytes. Accordingly, TNF-α-induced NLRP3 inflammasome activation was examined in 3T3-L1 adipocytes. Based on gene expression analysis, TNF-α upregulated the expression levels of gasdermin D (GSDMD, a component of inflammasome required for pyroptosis and IL-1β secretion [33]), caspase-1, and MCP-1; these expression levels were significantly ameliorated following METRNL pretreatment (Figure 7A). In contrast, METRNL treatment failed to protect against TNF-α-mediated NLRP3 protein upregulation (Figure 7B). Of note, IL-1β secretion was not detected in response to TNF-α treatment (Figure 7C), implying that despite the expression of inflammasome components, adipocytes may not be the primary site for canonical inflammasome activation. In conclusion, the exercise-mediated effect of METRNL, with respect to inhibition of NLRP3 inflammasome in adipose tissues, possibly involves modulation of macrophages and not adipocytes.

## 4. Discussion

Previous reports have shown that exercise training ameliorates obesity-induced inflammation, but studies assessing mechanisms responsible for these positive changes are lacking. In the present study, we explored the effect of exercise training and METRNL on NLRP3 inflammasome activation in adipose tissues to identify a potential target for the treatment of obesity-induced metabolic disorders. The main findings of this study are as follows: (1) exercise training ameliorates HFD-induced inflammasome expression in both SAT and VAT; (2) macrophages are reprogrammed by exercise to suppress NLRP3 inflammasome activation; (3) exercise training increases METRNL expression in various muscle depots; (4) METRNL treatment suppresses macrophage-induced IL-1β secretion via ERK and p38 MAPK activation. Collectively, these results indicate that METRNL mediates the crosstalk between muscle and adipose tissues during exercise to inhibit NLRP3 inflammasome activation and inflammation in adipose tissues.

Exercise is an effective treatment for metabolic disease, even in the absence of a marked reduction in body weight [34,35]. In some cases, a combination of exercise and diet has shown better effects in controlling metabolism and inflammation, along with weight loss [36]. Therefore, we designed the present animal study to compare the effect of exercise alone and in combination with dietary intervention. Along with changes in body weight, exercise and diet showed a synergistic effect on controlling systemic glucose tolerance and insulin sensitivity. Changes in HSL and ATGL mRNA expression levels in VAT support the marked changes observed in the fat mass in HFNCD+E mice. To our surprise, the inhibitory effect of exercise on NLRP3 inflammasome activation in adipose tissues and isolated BMDMs was comparable between the two groups, which is consistent with the results on macrophage infiltration. Additionally, we observed that the expression of inflammatory genes, such as TNF-α, NLRP3, and caspase-1, in VAT of HFNCD+E mice was at times higher than that in HFD+E mice, which may imply a stress response, owing to restriction in calorie. These results suggest that exercise plays a crucial role and is sufficient in regulating adipose tissue inflammation.

Reportedly, the effect of exercise training on metabolism is accompanied by a reduction in levels of both TLRs and proinflammatory cytokines [17,18]. Considering that TLR is involved in NLRP3 inflammasome activation [37], it can be postulated that exercise may also be involved in suppressing these pathways. Consistent with this notion, individualized exercise prescription has been reported to attenuate ASC gene expression in peripheral blood cells in obese individuals [38]. Animal studies have also shown that exercise suppresses NLRP3 inflammasome signaling in the aorta of obese mice [39], diabetic cardiomyopathy [40,41], and various brain regions, including the hippocampus [42], thus exerting protective effects against disease progression. However, the impact of exercise on adipose tissue NLRP3 inflammasome, as well as underlying mechanisms, remains poorly understood. In recent years, NLRP3 inflammasome has been identified as a crucial component of the mechanism underlying the development of systemic inflammation and progression of type 2 diabetes [13]. Notably, Vandanmagsar et al. first reported that NLRP3 inflammasome senses the lipotoxicity-associated increase in intracellular ceramide to induce caspase-1 cleavage in macrophages and adipose tissues [13]. Our results provide further evidence that exercise reverses HFD-induced inflammation and fibrosis in adipose tissues, partly due to suppression of NLRP3 inflammasome components and its stimulatory factors such as TXNIP and p62. For identifying the origin of inflammation, macrophages were examined ex vivo, and the results confirmed that exercise undoubtedly suppresses HFD-induced proinflammatory actions of macrophages. It is interesting to note that the basal level of the BMDMs isolated showed no difference in terms of NLRP3 inflammasome activation. However, when stimulated with LPS and ATP, both priming and activation steps were enhanced in BMDMs isolated from HFD mice, implying that the isolated macrophages maintain their metabolic status. We did not observe any change in systemic IL-1β levels, which is often upregulated in HFD-induced obese animal models [43,44]. However, changes in local production are expected to be more crucial, especially in regulating adipose tissue functions that lead to systemic effects on metabolism [45]. As supporting evidence, we observed a consistent reduction in NLRP3 inflammasome markers, including IL-1β, in adipose tissues and BMDMs. Secretory factors from macrophages seem to directly impact the regulation of adipocyte action, as evidenced by changes in adiponectin levels associated with adipocytes cultured under conditioned media, thereby indicating the macrophage-adipocyte crosstalk.

During exercise, peroxisome proliferator-activated receptor-gamma coactivator-1 alpha (PGC1α) mediates the release of METRNL from skeletal muscle into the blood, which plays an important role in browning and insulin sensitization of WAT via regulation of macrophage activity, thus suggesting that METRNL may help mediate exercise-induced protection against metabolic disorders [22]. Notably, intravenous administration of METRNL in non-obese diabetic mice delayed the onset of diabetes with altered cytokine secretion by immune cells, resulting in reduced pancreatic beta-cell destruction [46]. In another study, METRNL treatment in myoblasts was reported to stimulate glucose uptake via calcium-mediated AMPKα phosphorylation and the downstream p38 MAPK pathway, and intraperitoneal injection of GST-METRNL improved glucose intolerance in HFD mice, as well as in *db*/*db* mice [47], suggesting the therapeutic role of METRNL in metabolic disease. In the present study, our main objective was to delineate the anti-inflammatory role of METRNL with regard to the NLRP3 inflammasome. Our results revealed that METRNL pretreatment significantly downregulated IL-1β secretion in BMDMs, indicating a direct effect on macrophages. Moreover, this effect was partly mediated by activation of both ERK and p38 MAPK signaling. It is well known that ERK and p38 MAPK signaling play important roles in proinflammatory cytokine production and inflammation [48,49], and we have observed that both ERK and p38 inhibitors reduce the IL-1β secretion in BMDMs. However, it is apparent that these MAPKs also activate anti-inflammatory pathways (i.e., mitogen and stress-activated kinases, MSK) for preventing uncontrolled inflammation [50,51]. Since pharmacological inhibitors of ERK and p38 MAPK partly reversed the effect of METRNL on IL-1β secretion, it is likely that METRNL is involved in the anti-inflammatory role of this pathway. Consistent with our findings, METRNL knockout mice reportedly displayed dysregulated cytokine production and were highly susceptible to LPS in a sepsis model [52]. Moreover, recombinant METRNL treatment was found to alleviate inflammation in skeletal muscle and endothelial cells via AMPK or PPARδ signaling [23,53]. In addition, METRNL suppressed gene expression of NLRP3 inflammasome components in adipocytes, although no changes were observed at the protein level. Based on these reports, it can be speculated that METRNL is an effective target for suppressing NLRP3 inflammation in adipose tissues, and thus, has therapeutic potential for metabolic and inflammatory diseases.

METRNL has been previously shown to be secreted from whole muscle following exercise in vivo and by cultured myotubes upon electrical pulse stimulation in vitro [22,47], suggesting that exercise can increase the muscle contraction-induced secretion of METRNL. However, studies assessing the expression of skeletal muscle METRNL in response to exercise have been limited to mRNA levels or restricted to certain muscle types [54,55]. Herein, we examined three types of muscles (gastrocnemius, soleus, and quadriceps) and elucidated that mRNA levels of METRNL could be induced by exercise in all three depots, whereas protein levels were upregulated only in the gastrocnemius and quadriceps muscles in both exercise groups, with or without dietary intervention. Protein levels in the soleus muscle were not altered by HFD, exercise, or dietary intervention, which is in line with a previous report [56]. One explanation for this phenomenon could be related to the fact that soleus is a highly oxidative fiber type and may have reached its capacity in the expression of METRNL. In contrast, quadriceps and gastrocnemius having mixed fiber type have a greater capacity to undergo a switch from glycolytic toward oxidative type [57] and thus could be more sensitive in inducing METRNL expression in response to exercise. In addition, how METRNL expression and secretion are regulated by an acute bout of exercise is unknown since we observed the basal levels of METRNL after chronic exercise (i.e., sacrificed the day after the last bout of exercise). Therefore, which type of muscle contributes the most to the systemic effect of METRNL still needs further evaluation. Currently, limited reports on METRNL levels in overweight individuals and/or patients with obesity present contradictory results in terms of glucose intolerance [58,59,60,61], whereas exercise-induced METRNL secretion was observed to increase consistently [55,56]. One limitation of this study is the absence of data on plasma METRNL concentrations, as we were unable to determine the specificity of existing antibodies to measure METRNL concentration at the plasma level. However, based on the increased expression in skeletal muscle and evidence from previous reports, we assume that exercise training would have mediated the crosstalk between muscle and fat.

As METRNL is also reported to be expressed in macrophages and adipocytes [52,62], the possibility that local METRNL induction may have participated in regulating WAT function should be considered. Particularly, adipocyte METRNL has been shown to control insulin sensitivity via its local autocrine/paracrine action through the PPARγ pathway [62]. However, it has been reported that cold exposure induces METRNL expression in the adipose tissue but not the skeletal muscle, while downhill running exercise specifically induces METRNL expression in the skeletal muscle but not in the adipose tissue, emphasizing the fact that regulation of METRNL is tissue-selective depending upon the physiologic stimulus [22]. Therefore, METRNL as a myokine would have contributed the most in our animal model. Nonetheless, the involvement of METRNL release from other tissues is worth investigating in future studies.

## 5. Conclusions

Here we revealed that the anti-inflammatory effect of exercise was associated with METRNL induction in muscle and subsequent inhibition of NLRP3 inflammasome activation in macrophages, resulting in overall metabolic improvement. These results highlight the importance of METRNL in mediating the beneficial effects of exercise on metabolism. Further preclinical studies assessing the effects of METRNL administration would be crucial to elucidate its potential as a therapeutic agent for inflammation-related metabolic disorders.

## Data Availability

The data presented in this study are available in the figures and tables of this manuscript.

## Supplementary Materials

The following are available online at https://www.mdpi.com/article/10.3390/cells10123480/s1, Figure S1: Plasma IL-1β levels are unaltered by HFD or exercise, Figure S2: METRNL inhibits IL-1β secretion in BMDMs, Figure S3: U0126 and SB203580 inhibits the phosphorylation of ERK and p38 MAPK, Figure S4: ERK and p38 MAPK inhibition results in downregulation of IL-1β secretion in BMDMs.
