# Supplementary material for: Exercise Inhibits NLRP3 Inflammasome Activation in Obese Mice via the Anti-Inflammatory Effect of Meteorin-like
Source: Cells. 2021 Dec 9;10(12):3480. doi: 10.3390/cells10123480 (PMC8700724; doi:10.3390/cells10123480)
Supplement: Supplementary file 1 [file cells-10-03480-s001.zip › cells-1439119-supplementary.pdf]

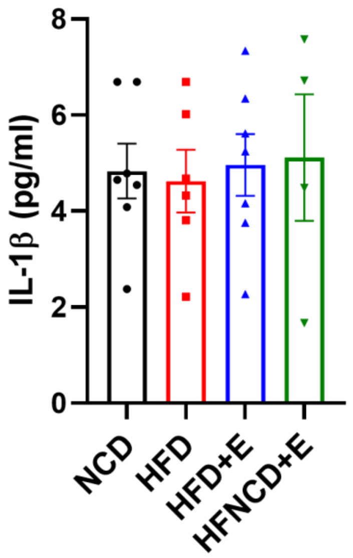


**Figure S1. Plasma IL-1β levels are unaltered by HFD or exercise**

Plasma was collected from NCD, HFD, HFD+E, and HFNCD+E mice to measure IL-1β levels using ELISA. Values are presented as mean ± SEM.

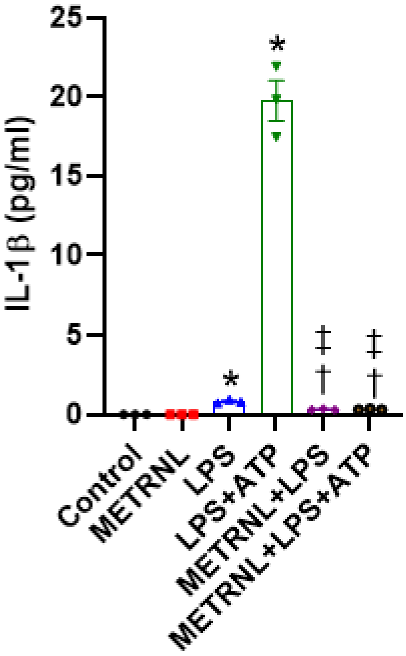


**Figure S2. METRNL inhibits IL-1β secretion in BMDMs**

BMDMs were pretreated with METRNL (100 ng/mL) for 1 h, followed by treatment with LPS (100 ng/mL) for 6 h. ATP (2 mM) was added during the last 30 min of treatment. The supernatant was collected, and IL-1β was measured using ELISA. Values are presented as mean ± SEM of triplicate experiments. * $P < 0.05$ compared with control, † $P < 0.05$ compared with LPS, ‡ $P < 0.05$ compared with LPS+ATP.

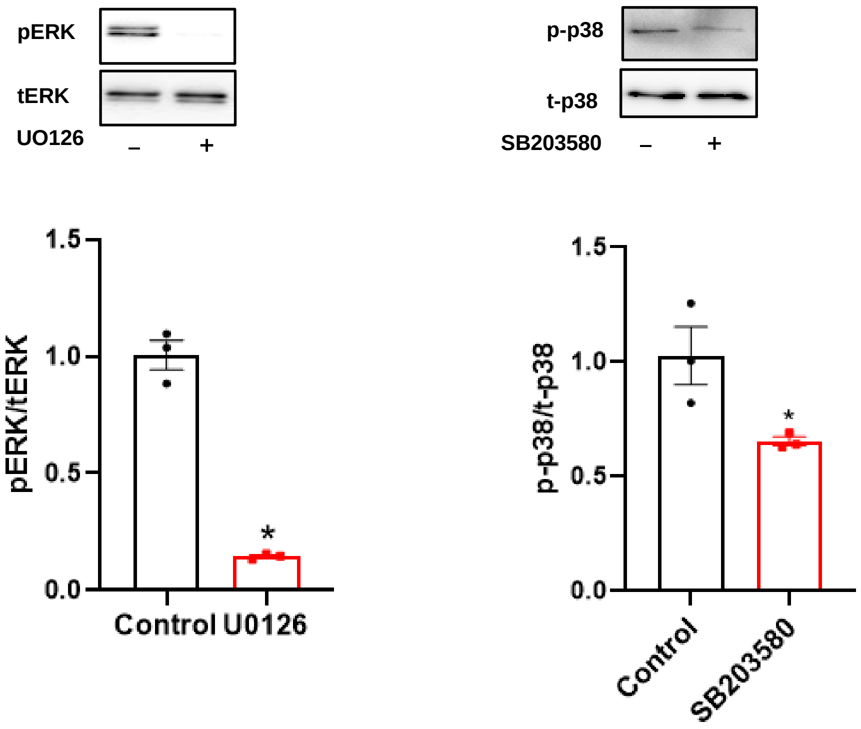


**Figure S3. U0126 and SB203580 inhibits the phosphorylation of ERK and p38 MAPK**

Representative Western blots and quantifications showing inhibition of ERK and p38 MAPK phosphorylation by 1 h treatment of ERK inhibitor (U0126, 30 μM) and p38 MAPK inhibitor (SB203580, 30 μM), respectively. BMDMs were isolated from 5-week-old male C57BL/6 mice. The phosphorylated protein levels were normalized to total protein levels (p = phosphorylated, t = total). Values are presented as mean ± SEM of triplicate experiments. * P < 0.05 compared with control.

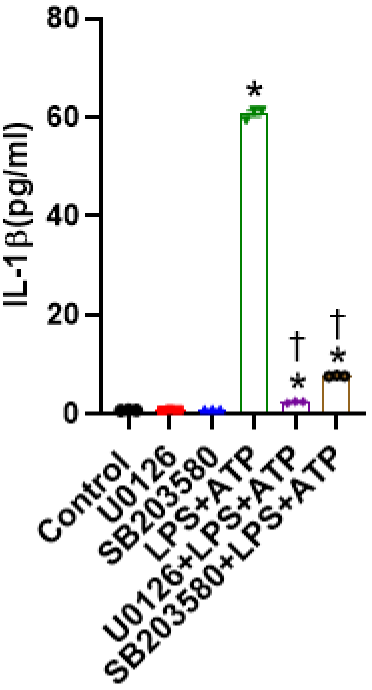


**Figure S4. ERK and p38 MAPK inhibition results in downregulation of IL-1β secretion in BMDMs**

BMDMs were pretreated with either ERK inhibitor (U0126, 30 μM) or p38 MAPK inhibitor (SB203580, 30 μM) for 1 h, followed by treatment with LPS (100 ng/mL) for 6 h. ATP (2 mM) was added during the last 30 min of treatment. The supernatant was collected, and IL-1β was measured using ELISA. Values are presented as mean ± SEM of triplicate experiments. * $P < 0.05$ compared with control, ł $P < 0.05$ compared with LPS+ATP.
